# Identification of Retinal Diseases Using Light Convolutional Neural Networks and Intrinsic Mode Function Technique

**DOI:** 10.3390/diagnostics16050773

**Published:** 2026-03-04

**Authors:** Preethi Kulkarni, Konda Srinivasa Reddy

**Affiliations:** School of Computer Science and Engineering, VIT-AP University, Amaravathi 522241, Andhra Pradesh, India

**Keywords:** fundus images, Light Convolutional Neural Network, Intrinsic Mode Function, Diabetic Retinopathy

## Abstract

**Background/Objectives**: Fundus imaging provides a detailed view of the interior surface of the eye and plays a crucial role in the early diagnosis of retinal diseases. However, automated interpretation of fundus images remains challenging due to variations in illumination, noise, and structural complexity. **Methods**: A novel hybrid model that integrates the Intrinsic Mode Function (IMF) filter, derived from Empirical Mode Decomposition (EMD), with a Light Convolutional Neural Network (LightCNN) for enhanced fundus image classification was proposed. The IMF filter effectively decomposes the input signal into intrinsic components, isolating high-frequency noise and preserving critical retinal patterns. These refined components are subsequently processed by the LightCNN architecture, which offers lightweight yet highly discriminative feature extraction and classification capabilities. **Results**: Experimental results on DIARETDB fundus datasets demonstrate that the proposed IMF + LightCNN model achieves 99.4% accuracy, 99.1% precision, 98.87% recall, and a 98.31 F1-score, significantly outperforming conventional CNN and ResNet-based models. **Conclusions**: Integrating advanced signal processing with lightweight deep learning improves both diagnostic accuracy and computational efficiency. This hybrid framework establishes a promising pathway for reliable and real-time clinical screening of retinal diseases.

## 1. Introduction

Diabetic Retinopathy (DR) is one of the major causes of blindness around the world. The diagnosis of these diseases in the early stages by fundus imaging has become a crucial part of modern-day ophthalmology. The fundus images capture the minute details of the retina, such as the optic disc, macula, and blood vessel patterns, enabling the detection of any abnormalities at an early level. But the analysis of these fundus images manually is a complex task and also leads to inter-observer variations in opinions. Also, the inconsistency in illumination, noise, and anatomical variations makes the task even more complex. All these factors emphasize the need for efficient and intelligent systems for the analysis and classification of these diseases. Recent advancements in the field of deep learning (DL) technologies have significantly improved the accuracy level for the analysis of various medical images. But most technologies consume a lot of processing resources and also need an enormous amount of data for training. Of these, the most interesting approach is the Intrinsic Mode Function (IMF), which is derived from the Empirical Mode Decomposition (EMD) technique. This IMF filter tends to enhance the high- and low-frequency components that provide more significant retinal structures and suppress the unwanted noise, thereby raising the discriminative capacity of the subsequent classifier. The LightCNN architecture has recently been one of the most efficient architectures for processing medical images by extracting meaningful features with much-reduced computational overhead. By combining IMF-based feature extraction with LightCNN’s lightweight architecture, this paper proposes a novel IMF+LightCNN framework that can classify retinal conditions with great accuracy while ensuring minimum latency. This hybrid approach bridges the gap between high-fidelity signal preprocessing and efficient deep learning-based classification, offering a scalable and clinically viable diagnostic solution.

### 1.1. Challenges

There are technical and clinical issues associated with the automated identification of retinal disorders from fundus images, which result in the limitations and unreliability of the current deep learning platforms for the diagnosis of such disorders. One of the main issues associated with the fundus images is the deficiency of uniform illumination and low contrast. These issues result in the degradation of minute details of the disorders like microaneurysms, exudates, and hemorrhages, and the noise or artefacts that may be produced in the process of capturing the image, such as blur, reflection, and background intensity variations. The traditional preprocessing technique that is less flexible in the sense that it tends to reduce important anatomical features along with the noise is fixed filtering and global histogram equalization. Because of these preprocessing techniques, the essential pattern in the retina is lost.

From a modelling perspective, many of the best practices in the field involve deep and complex ResNet-/DenseNet-based convolutional architectures. Although these architectures perform well, their deep architectures do in fact have complex computations that consume quite a bit of processing requirements in terms of memory utilization, processing, and many other factors.

Moreover, the lack of publicly available annotated datasets in the area of the retina adds a significant issue. Annotating medical images is a tedious task, and it needs expert ophthalmologists, and as such, the amount of available and balanced data is limited in this domain, hence the possibility of overfitting.

Lastly, a majority of existing models are classified as black-box models since they lack explainability in their predictions. The fact that they are not explainable is a hindrance to their adoption in medicine because medical professionals demand visual and structural explanations for their predictions in medicine.

### 1.2. Existing Approaches and Limitations

H. Lei et al. proposed an unsupervised domain adaptation method for joint segmentation of optic disc and optic cup in fundus images. By applying image synthesis and feature alignment, the authors were able to bridge the gap in segmentation performance resulting from domain variations across datasets [1]. W. Zhou et al. introduced a multi-scale adaptive adversarial learning method for unsupervised domain adaptation of fundus image segmentation models. The proposed approach uses adversarial learning to align both domains’ features together, while benefiting from multi-scale capabilities to improve segmentation outcomes and generalization across many fundus image datasets [2]. M. S. Junayed et al. presented CataractNet, which is a DL model for cataract detection automation from fundus images. The authors developed a detection model by combining convolutional neural networks (CNNs) with distinct feature extraction methods. The method offers an efficient complete automation method and tool for cataract detection which will be a benefit for early diagnosis/treatment of cataracts [3].

T. Palaniswamy et al.’s article describes the implementation of Internet of Things (IoT) and DL for DR diagnostics, which uses retinal fundus images. By integrating IoT with deep learning techniques, a smart system can be introduced to mitigate vision impairment for diabetic patient by identifying the early signs of Diabetic Retinopathy [4]. R. Rashid et al. authors explored the detection of retinitis pigmentosa using a deep learning model modelling SE-ResNet architecture which was trained using colour fundus images. This study focused on improving model performance and accuracy as well as the importance of detection of retinitis pigmentosa within the early stages of the disease while highlighting the difficulties in detecting the early stages of the disease and providing a novel method to improve model performance through data augmentation and transfer learning [5]. X. Li et al. proposed a rotation matching collaborative self-supervised learning framework. This method utilized unlabelled data while improving diagnostic accuracy by utilizing the symmetry of fundus images. Using self-supervised learning enabled the model to adjust and generalize better to different classifications of retinal disease include Diabetic Retinopathy [6]. M. Z. Atwany et al. focus on discussing the context of deep learning techniques and Diabetic Retinopathy classification. The authors discussed a variety of methods, including CNNs, for classifying Diabetic Retinopathy in fundus images. The authors discussed training challenges and discussed consequences of a lack of large, labeled data [7].

In the work by Y. Luo et al., a self-supervised fuzzy clustering network for retinal image classification is proposed to account for the problems posed by a shortage of labelled data in medical image analysis, applying unsupervised learning to perform segmentation and classification of retinal diseases. The fuzzy clustering strategy makes the model robust, even to noisy data [8]. J. Wang et al. outline prior-attention residual learning for discriminative COVID-19 screening for CT images. While this work does not apply directly to fundus images, it presents a way of using prior attention methods and residual learning to focus on medical imaging, which can be adapted to the retinal disease screening as well [9]. J. Civit-Masot et al. developed a dual machine-learning (ML) system to support glaucoma diagnosis. Combining simpler segmentation methods and machine learning will improve glaucoma diagnosis, particularly in differentiating the optic disc and cup by eye-to-eye evaluation [10].

F. Chen et al. established a dual-path and multi-scale enhanced attention network for retinal disease classification, which takes advantage of ultra-wide-field images. The inclusion of multi-scale attention mechanisms refers to the attention layers that learned a multi-scale consideration of the data and have a multi-scale fusion of retinal image inputs and report improvement in classification performance, which is especially useful for lesion detection and amongst other retinal abnormalities that can be confusing to trace using typical imaging approaches [11]. Y. Zhao et al. proposed a method for reconstructing retinal vascular network topology and classifying arteries and veins via dominant set clustering, this utilized a graph-based model to account for the analysis of the complicated retinal vasculature, giving insights into ocular conditions such as Diabetic Retinopathy and glaucoma [12]. X. Li et al. propose a self-supervised feature learning method on fabricated hyper-graph data and this method improved task accuracy by utilizing the labelled and unlabelled data within a mixed feature learning form while allowing generalization towards a number of retinal diseases [13]. E. Abdelmaksoud et al. offered an automated DR classification system developed to pick out multiple retinal lesions allowing detection of the presence of lesions through deep learning methods to produce image segmentation techniques with detection of the lesions to offer a production implementation of early diagnosis and grading of Diabetic Retinopathy through remote retinal fundus images [14]. Exudate regeneration was examined by M. Hussain et al. for automatic exudation [15].

E. O. Rodrigues et al. proposed ELEMENT, an approach to multi-modal retinal vessel segmentation which synergistically combines region-growing and machine learning techniques. This method produces a higher accuracy and better segmentation of the blood vessels in fundus photos through image processing and machine learning [16]. Y. Zong et al. presented a U-Net-based method to automatically segment hard exudates from fundus images with the aid of an inception module and residual connection. This method enhances the accuracy and efficiency of exudate segmentation, which is essential for the diagnosis of Diabetic Retinopathy, the leading cause of blindness amongst diabetic patients [17]. M. Shafiq et al. proposed Dual Eye-Feature Net, a dual-stream feature transfer framework for multi-modal ophthalmic image classification. This work aims to improve the classification of distinct retinal diseases by transferring features between modalities and using colour fundus and OCT image observations for better disease identification [18]. Atteia et al. [19] have implemented a model that retrieves features from two DL models, Resnet50 and Inceptionv3. The model follows feature extraction by using Resnet50—ResNet50 incorporates a range of convolutional filter sizes to expedite training and counteract the degradation challenge stemming from deeper network architectures. The Inception-v3 architecture is specifically tailored to visual classification to train fundus images.

Y. Wang et al. proposed a geometric correspondence-driven multimodal learning approach to ophthalmic imaging, developed in conjunction with the geometric mapping that can better align fundus and OCT data geometrically. Their method outperformed potential geometric divergences in traditional diagnostic approaches for a spectrum of ophthalmic diseases [20]. A. Ikram et al. provided an overview of fundus image-based strategies for classified detection and grading of Diabetic Retinopathy (DR), summarizing existing strategies, identifying challenges, and outlining ideal future actions and directions. They specifically noted advancements in the diagnoses of diabetic eye disease using deep learning-based models and needing very large, diverse datasets to help learning and improve the generalization of these models [21]. K. Aurangzeb et al. proposed a method for fundus image contrast enhancement via a modified version of particle swarm optimization, discovering significant improvements in performance for deep learning models utilizing retinal images of poor quality in detection of retinal disease [22]. Preethi.k et al. [23] proposed an OGFA(Optimized Graph Filter Algorithm) with CNN model to integrate the retinal patterns which correctly identified 11 labels of Diabetic Retinopathy and Macular Edema which showed high accuracy results. 

Recent developments in fundus image classification have been focused on deep learning, domain adaptation and their related segmentation techniques to identify retinal diseases of all various types and description. One of the challenges related to fundus image classification problems is that labelled datasets are not readily available especially in clinical environments where annotated microdatasets of fundus images for individual conditions are sparse. Studies demonstrated how unsupervised domain adaptation (UDA) approaches can direct deep learning (DL) models for various domain generalization improvements across different image datasets, when using limited or no labelled data. H. Lei et al. [1] and W. Zhou et al. [2] adopted new image synthesis and adversarial learning UDA-based techniques to ensure effective variability in deep neural networks to using different domains and data distributions for analysis. Variability to utilize different image datasets with limited labelled data is important for real world evaluations as obtaining sufficient labelled data is a difficult, time-consuming and costly task. Moreover, M. S. Junayed et al. [3] developed a deep learning-based tool, named CataractNet for automated detection of cataract in fundus images, promoting a theme towards automating complex diagnosis with AI enabled automated diagnosis systems.

A further promising area for classification of fundus images is the application of semi-supervised learning and self-supervised learning approaches to safely improve model accuracy in detecting Diabetic Retinopathy, so that the supervised learning approach is not the only constraining option when labelled data is limited. Low labelled data development [9] used self-supervised learning.

Proposed approaches in these studies could apply in classification of fundus images using multiple deep learning techniques, such as multi-scale adversarial learning, attention mechanisms, and semi-supervised learning to extract and classify high value features from fundus images. In the case of Diabetic Retinopathy, models would locate lesions and exudates by extracting the region of interest and then categorize the severity of the disease. Multi-modal learning frameworks, such as those which Y. Wang et al. [23] propose, can integrate heterogeneous image types (e.g., fundus images and OCT scans) to provide a more thorough diagnosis, allowing the models to categorize the classification as more accurate and robust. Methods such as dual-path networks and region-growing methods, such as those in F. Chen et al. [14] and E. O. Rodrigues et al. [19], can improve segmentation by analyzing retinal vessel patterns and lesions in scena to build more [robust] classifiers for Diabetic Retinopathy. These classifications could serve as the basis for multiple models, highlighting the strength of unsupervised and self-supervised learning, and could provide high-performance classification practices that could be employed in both clinical settings for intended usage by trained health professionals as well as potential real-time deployment for more abbreviated, expedited diagnosis of retinal diseases. A comparative summary of the literature survey is provided in Table 1.

#### Limitations of Existing Research Approaches

Although prior studies have demonstrated notable progress in retinal fundus image analysis, many of the existing approaches exhibit fundamental limitations when evaluated against real-world clinical and deployment requirements. A significant portion of the literature relies on **computationally heavy deep architectures** such as ResNet, dual-path attention networks, or multimodal frameworks, which, while accurate, are impractical for large-scale screening and resource-constrained environments. Furthermore, most approaches employ **fixed or heuristic preprocessing techniques** that lack adaptability to the non-stationary and non-linear characteristics of fundus images. As a result, subtle pathological structures, including microaneurysms and early-stage exudates, are often either suppressed or inadequately represented. Domain adaptation and self-supervised learning methods mitigate data scarcity to some extent; however, they predominantly focus on representation learning without explicitly enhancing disease-specific signal components. Additionally, several methods are limited to **single-disease detection** or depend on **multimodal data (e.g., fundus + OCT)**, restricting their applicability in fundus-only clinical screening workflows. These limitations collectively reduce robustness, generalization, interpretability, and deployment feasibility.

### 1.3. Motivation

The mentioned challenges motivate the development of a robust, adaptive, and computationally efficient framework capable of reliable retinal disease identification under real-world clinical conditions. In particular, there is a need for approaches that enhance disease-relevant retinal structures while suppressing acquisition-induced noise, without relying on rigid or handcrafted preprocessing techniques.

IMF decomposition, derived from EMD, provides an adaptive signal analysis framework that is well-suited for modelling non-stationary intensity variations and complex oscillatory structures inherent in fundus images. Unlike traditional filtering methods that rely on predefined frequency bases, IMF dynamically decomposes image data based on intrinsic characteristics, enabling preservation of subtle pathological patterns critical for early disease detection.

Concurrently, the increasing demand for deployable deep learning solutions highlights the importance of lightweight CNN architectures. LightCNN models are specifically designed to reduce computational complexity and memory usage while maintaining strong discriminative capability, making them suitable for large-scale screening, point-of-care diagnostics, and edge-based medical applications.

The central motivation of this work is to integrate adaptive signal decomposition with efficient deep learning by combining IMF-based enhancement and LightCNN-based classification. This fusion aims to overcome the individual limitations of conventional preprocessing and deep CNN models, resulting in an automated retinal disease classification system that is both accurate and clinically deployable.

### 1.4. Proposed Solution

Although there are considerable developments in deep learning-based classification systems for retinopathy diseases, most classification methods are based on computationally intensive convolutional networks and traditional image processing techniques with Gaussian filtering or histogram equalization processes. The proposed classification methods often struggle to effectively model **complex spatial variations and non-stationary intensity distributions** present in fundus images, particularly under varying illumination and image acquisition conditions. In addition, high-performance CNN models can be computationally intensive and therefore may not be appropriate for real-time detection in clinical settings.

However, to mitigate these shortcomings, a new hybrid approach is proposed that combines an Intrinsic Mode Function (IMF) decomposition method with a Light Convolutional Neural Network (LightCNN). Differently from the traditional preprocessing methods, the IMF technique, a result of Empirical Mode Decomposition, is an adaptive data-driven approach that separates a signal into components that are specific to a disease without the use of a set of prefixed basis functions. Also, the use of a LightCNN helps in effective feature learning despite a substantial reduction in the model size, which is indeed an advantage for a real-time application.

To the best of the authors’ knowledge, this is the first study to systematically combine IMF-based adaptive signal decomposition with a lightweight CNN architecture for retinal fundus image classification. The proposed IMF+LightCNN framework therefore represents a distinct methodological contribution that bridges signal processing and efficient deep learning for accurate and interpretable retinal disease diagnosis.

#### 1.4.1. Contributions

The overall contributions of the paper implored with the novelty of the design with light and IMF design solutions are indicated below:

***A novel IMF-based adaptive feature enhancement strategy*** is introduced for retinal fundus images, enabling effective separation of disease-relevant structural information from illumination and noise artefacts.

***A lightweight CNN architecture (LightCNN)*** is employed to achieve high classification accuracy while significantly reducing computational complexity, facilitating real-time clinical applicability.

***A unified IMF+LightCNN hybrid framework*** is proposed, combining signal decomposition and deep learning to improve robustness and generalization in retinal disease classification.

***An extensive experimental evaluation***, including multiple performance metrics and comparative analysis with state-of-the-art CNN and ResNet-based models, demonstrates the superiority of the proposed approach.

***Visual interpretability through enhanced result visualization***, including confusion matrices and performance curves, is provided to support clinical reliability and transparency.

#### 1.4.2. Effectiveness of the Proposed IMF+LCNN Framework

The proposed method enhances disease-relevant representations prior to learning by employing IMF-based adaptive decomposition.

IMF decomposition differs fundamentally from commonly used wavelet- or Fourier-based approaches by adaptively extracting non-stationary and non-linear oscillatory components directly from the input image. This enables selective emphasis of mid-frequency pathological patterns associated with microaneurysms, hemorrhages, and exudates, while suppressing background texture and illumination artefacts. As a result, clinically meaningful structures are explicitly preserved rather than implicitly learned by deep layers alone.

The subsequent LightCNN architecture complements this signal-aware preprocessing by enabling accurate classification with significantly reduced computational complexity. In contrast to recent lightweight CNN approaches that rely solely on spatial feature learning, the proposed integration of IMF-based enhancement and LightCNN achieves improved robustness, generalization, and interpretability under varying imaging conditions.

While contemporary studies explore self-supervised learning, adversarial training, or multimodal data fusion, the present work deliberately focuses on supervised fundus image analysis using adaptive signal decomposition and a lightweight CNN backbone. This design choice prioritizes clinical deployability, simplicity, and scalability, thereby advancing the state-of-the-art toward practical large-scale retinal screening solutions.

## 2. Materials and Methods

The upcoming experiment on fundus image classification enhances automated diagnostic systems for Diabetic Retinopathy. This requires multi-modal deep learning, unsupervised domain adaptation, and semi-supervised learning. These approaches enhance model performance on small, labelled samples and poor-quality images. It will be capable of classifying and differentiating critical sections: hemorrhages, microaneurysms, exudates, and blood vessels indicating eye disorders.

The approach integrates the best algorithms: GANs for generating images, attention mechanisms for extracting relevant features, and multi-scale learning techniques for generalization and accuracy. Unsupervised machine learning techniques will learn useful features from the unlabelled images of the fundus of the eye. The technique will allow the classifier to generalize, even without any labelled images. UDA techniques will make the system suitable for various sets and environments, which will enhance generalization and applicability in practice.

### 2.1. Data Preprocessing and Augmentation

Reliable retinal disease classification requires robust preprocessing and augmentation strategies to address variations in image quality, illumination, and acquisition conditions commonly encountered in fundus imaging. In this work, a carefully designed preprocessing and augmentation pipeline is employed to enhance clinically relevant retinal structures while preserving pathological integrity.

#### 2.1.1. Image Normalization

All input fundus images are first resized to a uniform spatial resolution of 224×224 pixels to ensure compatibility with the LightCNN architecture. Pixel intensity normalization is then applied to scale image values into a consistent range, reducing inter-image variability and stabilizing network training. The normalization process is defined as

$$I_{\mathrm{norm}}=\frac{I-I_{min}}{I_{max}-I_{min}}$$

where I represents the original pixel intensity, and $I_{min}$ and $I_{max}$ denote the minimum and maximum pixel values, respectively. For RGB images, grayscale conversion is performed using a standard luminance-preserving transformation to focus the model on structural and intensity-based retinal features.

##### Contrast Enhancement Using CLAHE

To improve the visibility of fine retinal structures such as blood vessels, microaneurysms, and exudates, Contrast Limited Adaptive Histogram Equalization (CLAHE) is applied. CLAHE enhances local contrast while preventing over-amplification of noise. The following parameters are used consistently across all experiments:

**Clip limit**: 2.0
**Tile grid size**: 8×8
**Interpolation**: Bilinear

These parameters were empirically selected to balance contrast enhancement and noise suppression. Clinically, CLAHE is valid because it mimics ophthalmologists’ practice of adjusting local contrast to better visualize subtle lesions during manual examination, without altering the underlying anatomical structure.

#### 2.1.2. IMF-Based Feature Enhancement

After contrast enhancement, fundus image analysis involves Intrinsic Mode Function (IMF) decomposition based on Empirical Mode Decomposition. IMF decomposition breaks down each image adaptively into a series of intrinsic oscillations that depict different spatial frequencies. The high spatial frequency IMF is concentrated on small pathological details like microaneurysms and hemorrhages, whereas the lower spatial frequency IMF captures the global illumination trend. Some IMF decomposition results are chosen and combined to remove background artefacts and highlight relevant disease structures before deep feature extraction.

#### 2.1.3. Data Augmentation Strategy

Extensive but clinically constrained data augmentation is used in training in order to improve generalization and counter overfitting because of a lack of annotated data in the medical domain. Each operation in the data augmentation process is performed with a certain probability.

The augmentation parameters are as follows:

**Rotation**: Random rotation within the range of −15° to +15°.

**Horizontal flipping**: Applied with a probability of 0.5.

**Vertical flipping**: Applied with a probability of 0.3.

**Scaling (Zoom)**: Random geometric scaling in the range of 0.9 to 1.1 applied prior to resizing.

**Translation**: Horizontal and vertical shifts up to ±10% of image dimensions.

**Overall augmentation probability**: 0.7 per image.

Each transformation is applied independently based on its probability, ensuring diverse yet realistic variations.

**Clinical Validity of Augmentation Operations**

All the operations of the augmentation are carefully selected to make the outcome realistic. The rotation of a small angle helps to mimic the movement of the head and the position of the camera during the acquisition of the fundus. Flipping the image horizontally and vertically helps to make the image realistic due to the symmetrical nature of the retinal components. When the image is scaled and translated moderately, it helps to make the retinal positions and distances from the camera realistic. It is important to note that the image will not be subjected to extreme transformation.

**Figure 1:**
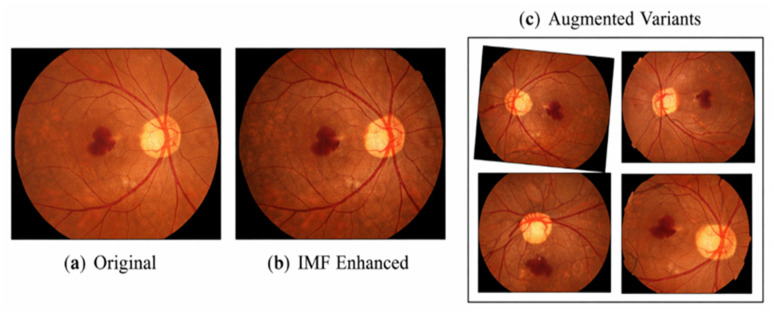
IMF-based enhancement and geometric augmentation of retinal fundus images.

Figure 1 illustrates representative examples of the preprocessing and augmentation stages:

(a) Original fundus image.
(b) IMF-decomposed and recombined feature-enhanced image.
(c) Augmented variants including rotation, flipping, and scaling.

This visual comparison highlights how the proposed pipeline enhances retinal structures while generating realistic data diversity for robust model training.

### 2.2. Proposed Approach (IMF+LCNN)

#### 2.2.1. Empirical Mode Decomposition (EMD): Formal Definition

Fundus images are characterized by **non-linear and non-stationary intensity variations** due to uneven illumination, vessel curvature, and localized pathological changes. Conventional frequency-domain techniques such as Fourier or wavelet transforms rely on predefined basis functions and therefore lack adaptability to such characteristics. To overcome this limitation, the proposed framework employs **Empirical Mode Decomposition (EMD)**, a fully data-driven signal decomposition technique. Given an input fundus image I(x,y), EMD decomposes the signal into a finite set of **Intrinsic Mode Functions (IMFs)** and a residual component as

(1) $$I(x,y)=\sum_{k=1}^{K}{IMF}_{k}(x,y)+R(x,y)$$

where

${\mathrm{IMF}}_{k}(x,y)$ represents the k-th Intrinsic Mode Function,

K is the total number of extracted IMFs,

R(x,y) denotes the residual trend containing low-frequency background information.

Each IMF captures oscillatory modes at distinct spatial frequency scales, ranging from fine-grained textures to coarse illumination patterns.

#### 2.2.2. Mathematical Conditions for an Intrinsic Mode Function

1. For a decomposed component to qualify as an IMF, it must satisfy **both of the following conditions**:
2. **Extrema–Zero Crossing Condition**

The number of extrema and the number of zero crossings must either be equal or differ by at most one:

(2) $$|N_{\mathrm{extrema}}-N_{\mathrm{zero}-\mathrm{crossings}}|\;\leq 1$$

3. Local Mean Condition

At any point, the mean value of the upper envelope U(x,y) and lower envelope L(x,y) must be zero:

(3) $$m(x,y)=\frac{U(x,y)+L(x,y)}{2}=0$$

4. These conditions ensure that each IMF represents a **well-behaved, narrow-band oscillatory mode**, making it suitable for frequency-aware feature analysis.

#### 2.2.3. Sifting Process and IMF Extraction

The IMF extraction follows an iterative **sifting process**, summarized as

1. Identify all local maxima and minima of I(x,y).
2. Interpolate maxima to form the upper envelope U(x,y).
3. Interpolate minima to form the lower envelope L(x,y).
4. Compute the local mean:
  (4) $$m(x,y)=\frac{U(x,y)+L(x,y)}{2}$$
5. Extract the detail component:
  (5) h(x,y)=I(x,y)−m(x,y)
6. Repeat steps 1–5 until IMF conditions are satisfied.
7. Subtract the IMF and repeat the process on the residual.
8. This adaptive process continues until the residual becomes monotonic.

#### 2.2.4. Selection of Relevant IMF Components

**Figure 2:**
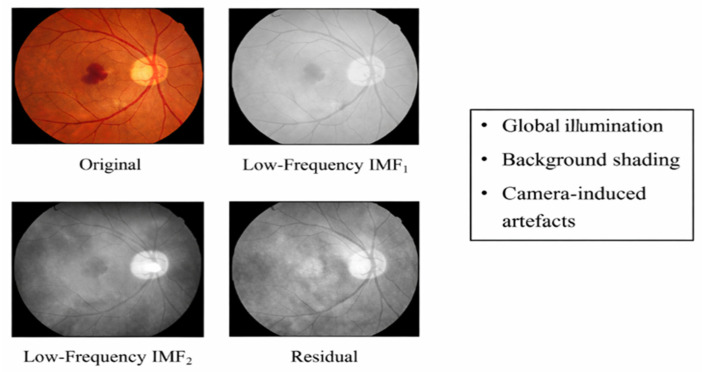
Low-frequency illumination and background Vvariations.

However, not all Intrinsic Mode Function (IMF) terms are of equal importance for an efficient disease discrimination task on retinal fundus images, since every IMF corresponds to a particular band of spatial frequencies, which carry varying importance levels of disease information. The higher-frequency IMFs (IMF_1_–IMF_2_) as shown in Figure 2 mainly preserve noise information and artefacts induced by the sensor, as well as very small-scale intensity variations not meaningful to represent disease information, which if included, may cause ill effects on disease classification performance. On the contrary, the medium frequency IMFs (IMF_3_–IMF_5_) contain the most important disease information of the retina, such as microaneurysms, exudates, and hemorrhages, as well as the boundaries of blood vessels. The above disease information appears as local intensity variations as well as structural changes, which match closely with the mid-range oscillations captured by EMD, thereby providing critical signals for efficient feature discrimination through the selection of medium IMFs, while discarding the noise-containing high IMFs as well as non-significant background information.

Based on empirical analysis, the proposed method **retains mid-frequency IMF components** and suppresses both high-frequency noise-dominated IMFs and low-frequency background components. The reconstructed enhanced image is expressed as

(6) $$I_{\mathrm{IMF}}(x,y)=\sum_{k=p}^{q}{IMF}_{k}(x,y)$$

where p and q correspond to the selected mid-frequency IMF indices.

#### 2.2.5. Clinical Interpretation of IMF Frequency Bands and Integration with Light CNN

Intrinsic Mode Function (IMF) extraction arises from the spatial and structural characteristics of retinal pathologies observed in fundus images. Clinically relevant abnormalities such as microaneurysms, hemorrhages, and exudates typically manifest as localized intensity variations and abrupt spatial transitions. These pathological features correspond to mid-frequency components in the spatial domain, making them well-represented by higher-order IMFs obtained through Empirical Mode Decomposition (EMD).

In contrast, low-frequency IMFs primarily capture slowly varying illumination trends caused by non-uniform lighting, vignetting, and camera-induced shading effects. These components contribute little to no diagnostic content and often suppress lesion contrast. High-frequency IMFs, on the other hand, are dominated by sensor noise and fine-grained texture variations that are not clinically meaningful. By selectively retaining mid-frequency IMFs and discarding low- and high-frequency components, the proposed approach enhances disease-related structural details while suppressing illumination artefacts and noise.

The IMF-enhanced image generated through this frequency-aware reconstruction is subsequently used as input to the Light Convolutional Neural Network (LightCNN) classifier. Unlike conventional CNNs that must implicitly learn to disentangle pathological structures from background artefacts, the proposed framework performs explicit structural separation prior to deep learning. This preprocessing step provides the LightCNN with a cleaner and more informative input space, allowing it to focus on learning discriminative retinal features rather than compensating for acquisition-related variations.

As a result, the LightCNN exhibits faster convergence during training and improved generalization performance, as the risk of overfitting to noise and illumination artefacts is significantly reduced. Moreover, the explicit separation of structural information through IMF decomposition enhances the interpretability of the learned representations, as the network responses are more directly associated with clinically meaningful retinal structures.

#### 2.2.6. Workflow of the Proposed IMF-Based Feature Learning

**Figure 3:**
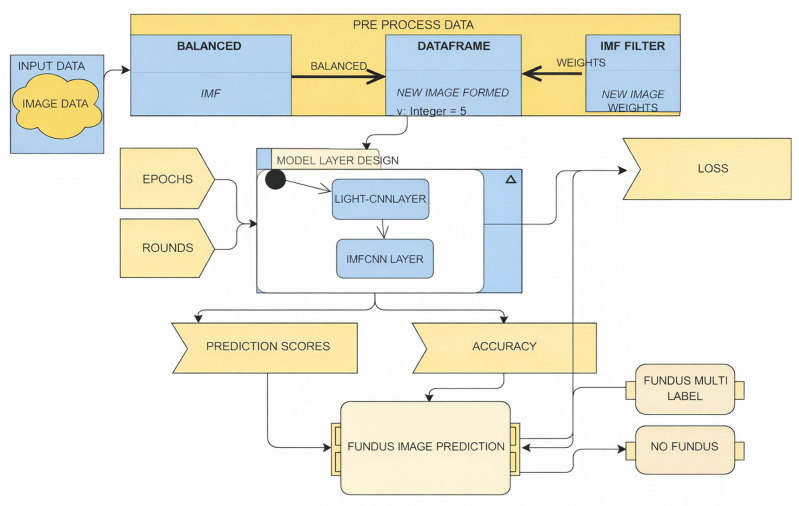
Architecture of IMF and LCNN technique.

Figure 3 shows the overall process of the suggested retinal disease classification system. The system takes fundus images as input and subjects them to some preprocessing and contrast enhancement steps for ensuring the uniformity of the images. The resulting contrast-enhanced images are then processed using the Empirical Mode Decomposition technique, which delivers several Intrinsic Mode Functions. The mid-frequency components of the IMFs are chosen and combined for the creation of an IMF-enhanced image that shows the retinal areas affected by the disease while removing the irrelevant illumination effects and noises. The resulting image is processed with the LightCNN architecture for classification. Figure 3 shows the classification process and does not include self-supervised learning, adversarial learning, and the adaptation learning process for better clarity and understanding. In the proposed approach, the decomposition using Intrinsic Mode Function (IMF) is strictly used as a signal-level processing technique for preprocessing and enhancement before the classification is achieved by using a Light Convolutional Neural Network (LightCNN). The step of decomposition by IMF is directly applied to the input fundus images to eliminate noises as well as illumination effects before moving to deep learning.

After processing and contrast enhancement, every fundus image decomposed using Empirical Mode Decomposition into several IMF components, among which mid-frequency IMF components (IMF_3_–IMF_5_) have been selected through empirical observation, which contain the necessary microscopic and macroscopic features of the retina in terms of microaneurysms, exudates, hemorrhages, and vascular margins, while high-frequency and low-frequency IMF components, comprising the majority of noise and background light, have been eliminated. An IMF-enriched image is reconstituted through IMF recombination and directly fed into the LightCNN classifier.

The LightCNN structure is capable of learning discriminative spatial features from the amplified representation by using convolutional layers and max feature map operations followed by fully connected layers for classification. Notably, for the experimental framework in this study, neither self-learning approaches nor adversarial learning nor domain adaptation is incorporated. These aspects are outside the optimization and learning process and are posited in future studies or extensions as possible avenues for future work and development. The critical move of decoupling and demarcating adaptation and learning for signal enhancement from feature learning in the proposed approach has alleviated learning complexity and facilitated convergence and learning stability while promoting adaptation and enhancement in generalization capability even in divergent sizes of learning samples and datasets.

Algorithm 1 summarizes the complete IMF-filtered LightCNN classification procedure illustrated in Figure 3. The IMF decomposition is strictly confined to the preprocessing stage for illumination and noise suppression, while the LightCNN-based model layer is responsible for feature learning and classification. This separation of signal enhancement and deep learning avoids redundancy in feature learning and contributes to stable convergence and improved generalization.
**Algorithm 1: IMF-Based Adaptive Feature Enhancement and LightCNN Classification****IMF Algorithm****Input**
Fundus image dataset $D=\left\{I_{1},I_{2},\ldots ,I_{N}\right\}$ with corresponding class labels $L=\{y_{1},y_{2},\ldots ,y_{N}\}$
**Output**
Predicted retinal disease classes $P=\left\{{\hat{y}}_{1},{\hat{y}}_{2},\ldots ,{\hat{y}}_{N}\right\}$ with confidence scores   
**Step 1: Image Preprocessing**
For each fundus image $I_{i}\in D$:
○Resize to 224×224○Convert RGB to grayscale using:
$I_{g}=0.2989R+0.5870G+0.1140B\;$○Normalize pixel values:
$$I_{\mathrm{norm}}=\frac{I_{g}-I_{min}}{I_{max}-I_{min}}$$
   **Step 2: Contrast Enhancement**
2.Apply CLAHE to $I_{\mathrm{norm}}$ using:
○Clip limit = 2.0○Tile grid size = 8×8
   **Step 3: Empirical Mode Decomposition (EMD)**
3.Perform EMD on the enhanced image:$I(x,y)=\sum_{k=1}^{K}{\mathrm{IMF}}_{k}(x,y)+R(x,y)$4.Extract IMF components using the sifting process until IMF conditions are satisfied:
○Zero-crossings ≈ extrema○Mean of upper and lower envelopes ≈ 0
   **Step 4: IMF Component Selection (Critical Update)**
5.Select **mid-frequency IMF components** based on empirical frequency–pathology analysis:
○Retain: ${\mathrm{IMF}}_{3}$ to ${\mathrm{IMF}}_{5}$○Discard:
■High-frequency IMFs (noise, artefacts)■Low-frequency IMFs + residual (illumination, background shading)
6.Reconstruct the IMF-enhanced image: $I_{\mathrm{IMF}}=\sum_{k=3}^{5}{\mathrm{IMF}}_{k}(x,y)$

   **Step 5: Data Augmentation (Training Only)**
7.Apply clinically valid augmentations with probability 0.7:
○Rotation: $\pm 15^{\circ }$○Horizontal flip (*p* = 0.5)○Vertical flip (*p* = 0.3)○Scaling: 0.9–1.1○Translation: ±10%
   **Step 6: LightCNN Feature Learning**
8.Feed $I_{\mathrm{IMF}}$ into the LightCNN architecture:
○Convolution + Max-Feature-Map layers ○Batch normalization and dropout○Global average pooling
9.Learn hierarchical spatial features emphasizing:
○Lesions○Vessel boundaries○Optic disc structures
   **Step 7: Classification**
10.Apply fully connected layer with Softmax activation:
$${\hat{y}}_{i}=arg\;max\left(\mathrm{Softmax}(Wf_{i}+b)\right)$$
   **Step 8: Model Training**
11.Split dataset into
Training (70%)Validation (15%)Testing (15%)
12.Train using
Optimizer: AdamLoss: Categorical Cross-EntropyEarly stopping on validation loss
   **Step 9: Evaluation**
13.Compute performance metrics:
AccuracyPrecisionRecallF1-scoreConfusion matrixROC-AUC
   **Return**


### 2.3. Deep Learning Layer Design Using IMF

The IMF (Image Feature Mapping) Layer Architecture utilizing dense layers is a complex methodology focused only on enhancing the feature extraction and classification process, notably for medical image analysis tasks such as fundus image classification. The architecture begins by extracting low- and mid-level features from fundus images through traditional image processing methods or DL through CNN. The images were then extracted of the features including, the salient structures that could be important and processed through the IMF layer, to transform the features from the image feature extraction, with dense layers capturing local and global relationships and identifying ways to refine the features as well. Once the features are extracted through their respective processes, the IMF architecture converts the multi-dimensional feature maps into a one-dimensional vector to be processed through the dense layers. The first layer (dense layer), takes the flattened vector and applies a weight matrix with bias and the first non-linear activation function (ReLU—Rectified Linear Unit); through successive dense layers, the layers gradually are able to enhance further refine the features and enable the model learn to represent more abstract and more complex representations from the features. As a result of the model’s hierarchical learning, the model is further able to capture the gradients that identify subtle differences amongst different time intervals for Diabetic Retinopathy within the trainable data set. Each subsequent layer applies non-linear activations with their own biases and weight matrix to produce the representation of the features; see the first dense layer to the last dense layer.

Outputs from dense layers are generally directed to final classification layers, and the layers can be classified as either binary or multi-class, depending on the task. Binary classification tasks incorporate a sigmoid activation function, and multi-class classification tasks use the softmax function to normalize raw outputs into class probabilities, which allows the network to classify the input image into classes. Dropout layers may be interspersed throughout the network to help generalize the model as part of the dense architecture to prevent overfitting with small datasets or noisy medical images. In medical image analysis, overfitting is especially critical as it is paramount to generalize the model to new data that is unseen for accurate diagnosis. The ultimate objective of the IMF layer architecture is to provide an end-to-end learning process through dense layers. It can automate the learning of complex representations of input data and avoid manual feature extraction processes. The performance of this IMF-Layer architecture is very useful in feature variation and for those that are very complex in medical imaging tasks, such as fundus image classification. Dense layers allow the model to learn hierarchical relations and point out distinctions within retinal images that will be significant in detecting disease correctly. Such architecture is suitable for all types of images, thus offering great flexibility to deal with different types of medical imaging situations by supplying reliable performance against datasets that are varied and noisy. In brief, a dense layer IMF architecture is efficient, scalable in design to be availed for fundus image classification while allowing a network to extract only discriminative features and classifications, with less human intervention. The deep learning model’s ability to learn hierarchical, and domain agnostic features within the retina, along with the third dense layer’s capability of refining the patterns extracted by previous two layers, is a very useful component for automated disease detection. This type of process is a large advancement in the foray into medical imaging, and more significantly, with Diabetic Retinopathy, where earlier detections can greatly augment initial treatment options.

### 2.4. Hypertuning

Hyperparameter tuning is essential to achieve optimal performance of DL models and is important in areas like fundus image classification which entails identifying eye diseases such as Diabetic Retinopathy and cataracts from retinal of byproducts of medical devices. For IMF models the most relevant hyperparameters to tune are the learning rate, batch size, number of convolutional layers, kernel size, dropout rate, and training epochs. Learning rate is the most important of these since it establishes the size of the step taken when updating the model weights and therefore influences both the speed and stability of convergence. A proper choice made in learning rate will help the model converge much faster whilst not overshooting optimal values. The batch size determines how often the model parameters are updated. A small batch size will offer to update the model parameters more frequently than a large batch which smooths out the gradient update over more samples and will more likely offer enhanced generalization. The kernel size and number of convolutional layers will affect the model’s ability to extract features from hierarchical and spatial fundus images. If model hyper-parameters are selected correctly, the model should be able to adequately capture both fine and coarse structures. A key regularization hyper-parameter is the dropout rate due to randomly turning off neurones during training to help prevent overfitting. This is important for medical imaging where labelled data may be limited and overfitting may occur. The number of epochs or iterations of training will also have to be carefully chosen to avoid underfitting or overfitting. Early stopping strategies are commonly utilized to stop training, once improvement on validation data has ceased. In the case of model dense layer models, model hyper-parameter tuning shifts towards the fully connected dense layers that follow feature extraction. Primary hyper-parameters include the following also, number of dense layers, number of units per layer, activation functions, optimisers and learning rate decay schedule. Together the depth and width of the dense layers will dictate the effectiveness of a model’s ability to learn complex patterns from the extracted features. When considering the number of units, performance can be improved by increasing the size; however, this also increases the risk of overfitting.

Activation functions such as the ReLU function add non-linearity to a network, which is required for models to create complex patterns. Choosing the right activation function is very important when looking to optimize the learning process. Can you use alternative options—adaptive optimization algorithms come in handy for optimizing model performance with decay of learning rates. By using a decay of the learning rate, the training starts with a large change in weight, then progressively decrease it to allow the model to settle into a figure that is closer to optimal. Batch normalization is frequently used to stabilize training with the dissertation that it will normalize the output of each layer. This normalization can lead to quicker convergence of the model or training time or better accuracy, but usually just for deeper networks. There are multiple models such as, Grid search—is simply tests all the combinations of pre-defined hyperparameters; however, this is computationally expensive. Random search is tests a random of combinations, usually just as improvement but again only with a reduced computational cost. Bayesian optimization will use a model to model the hyperparameters’ performance and will choose the next best configuration based on previous evaluations. These processes can be supported with advanced tools and libraries that automatically tune the model. These types of tools will rate your model based on performance evaluation metrics to find the most effective—hyperparameters settings. Cross-validation is also applied to measure how well a model generalizes with different subsets of dataset; accurate performance on unseen past data can lead to a confident model. Hyper-parameters involve some creativity in finding the right settings for the model to emulate the best fit.

### 2.5. Experimental Setup

The experimental design in classifying fundus images of the retina is conceptualized to support not only model training but also model assessment in a reliable and fair manner despite differences in dataset scale. Apart from identifying suitable datasets and training processes, a significant focus lies in model validation with respect to small, medium, and large datasets, a practice that plays a crucial role in understanding model generalization and viability.

#### 2.5.1. Dataset Description and Scale-Wise Utilization

The experiments are performed using a public fundus image dataset called DIARETDB, a commonly used standard in the field of fundus image analysis. The dataset includes high-resolution, colourful fundus images annotated with various retinal pathology marks, such as microaneurysms, hemorrhages, and exudates.

To evaluate the robustness, scalability, and generalization capability of the proposed IMF–LightCNN framework, the dataset is utilized at three different scales: a **small-scale** subset (~5000 images) representing data-scarce clinical scenarios, a **medium-scale** subset (~20,000 images) reflecting typical institutional datasets, and a **large-scale** subset (~50,000 images) simulating population-level screening conditions.

For all scales, the data are partitioned using a **stratified 70%/15%/15% split** for training, validation, and testing, respectively, to preserve class distribution and mitigate class imbalance bias.

#### 2.5.2. Model Architecture and Training Configuration

This model combines IMF-based adaptive feature enhancement with a **Light Convolutional Neural Network (LightCNN)** for classification, as detailed in Section 2 and illustrated in Figure 3. The LightCNN architecture is employed without structural modification.

Model training is performed using the **Adam optimizer** with an adaptive learning rate. The key training hyperparameters are summarized as follows:

- **Batch size**: 32
- **Number of epochs**: 50
- **Initial learning rate**: $1\times 10^{-4}$
- **Loss function**: Categorical cross-entropy
- **Regularization**: Dropout and early stopping based on validation loss

Hyperparameter tuning is conducted using **grid search and Keras Tuner**, particularly for learning rate, dropout ratio, and network depth, to ensure optimal performance across all dataset scales.

#### 2.5.3. Hardware and Software Environment

Each and every experiment is conducted on a GPU-accelerated computational setup with TensorFlow and the support of CUDA. This is crucial for effective training processes on mid- and large-scale datasets, considering the fact that the size of the fundus images plays a substantial role in increasing the computational requirements for the overall process.

#### 2.5.4. Evaluation Protocol and Performance Metrics

A thorough assessment of model quality is achieved by testing the model on the independent test data for a given scale within each dataset. Model quality is evaluated using a number of criteria that are important in the clinical field:

- **Accuracy.**
- **Precision.**
- **Recall (sensitivity).**
- **F1-Score.**
- **Area Under the ROC Curve (AUC-ROC).**
- **Dice Similarity Coefficient (DSC) (for structure-level evaluation).**
- **Confusion Matrix.**

These factors, taken together, give an estimate of the reliability of classification, discrimination of classes, and robustness to imbalances in classes. The use of confusion matrices and ROC curves adds to this interpretability. Cross-validation is utilized during the training process to be able to perform properly on any sample of data that could represent different subsets of images.

## 3. Results

### 3.1. Ablation Study and Component-Wise Contribution Analysis

In this work, an ablation study was conducted to quantitatively assess the individual and combined contributions of the IMF-based preprocessing and the lightweight CNN (LCNN) architecture within the proposed IMF+LCNN framework. Each component was systematically removed or replaced while keeping the remaining settings unchanged.

When the LightCNN was trained directly on raw fundus images without IMF-based enhancement, a noticeable degradation in testing accuracy and recall was observed, particularly in cases involving low-contrast lesions and early-stage pathological signs. This decline indicates that raw fundus images contain illumination variations and background artefacts that adversely affect discriminative feature learning.

Conversely, applying IMF-based preprocessing followed by a conventional CNN resulted in moderate performance improvement over the baseline CNN. This confirms that IMF decomposition enhances signal quality by suppressing low-frequency illumination noise and emphasizing lesion-related structures. However, this configuration showed limited scalability and weaker generalization on medium- and large-scale datasets, suggesting that IMF enhancement alone is insufficient for optimal performance.

The full IMF+LCNN configuration consistently achieved superior performance across all evaluation metrics. These results demonstrate that IMF-based adaptive signal decomposition primarily contributes to **noise suppression, illumination normalization, and lesion contrast enhancement**, while the lightweight CNN architecture contributes to **efficient feature learning, reduced overfitting, and improved scalability**. The complementary integration of these components is therefore critical to the performance gains reported in this study.

### 3.2. Training Analysis

At the training stage, the deep learning (DL) model for fundus image classification and segmentation is trained with a dataset containing more than 50,000 images. Large datasets allow the model to learn different features and identify varied patterns that will help distinguish the eye diseases. Typically, dataset is split into 80–20% sets as train (80%), validation (10%), and testing (10%), for better evaluation of the model. In order to increase robustness and accommodate variability in the image quality and resolution, data must be augmented. To further emphasize important retinal features Contrast Enhancement methodologies such as histogram Equalization or CLAHE are used to better emphasize and highlight main retinal features and thus improving the accuracy of classification and segmentation.

**Figure 4:**
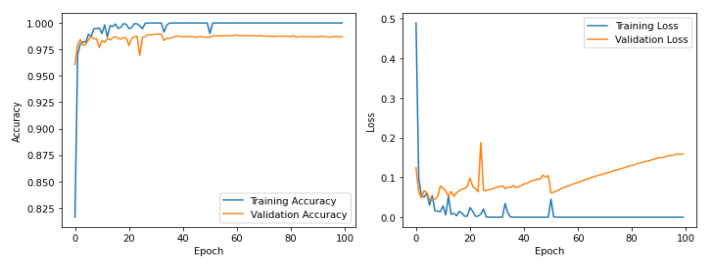
Training and validation accuracy and loss over epochs with 50 K fundus images.

Figure 4 illustrates the training and validation accuracy and loss curves over multiple epochs. The rapid reduction in both training and validation loss during the early epochs indicates efficient feature learning, while the close alignment between the two curves suggests minimal overfitting. The stabilization of accuracy values in later epochs demonstrates convergence and stable learning behaviour, validating the effectiveness of the lightweight architecture in handling large-scale datasets without excessive model complexity.

### 3.3. Testing Analysis

Once the training process is complete, the model enters testing mode, where its performance is evaluated against an independent test set of fundus images that have no previous interaction with the training process. The test data set is also processed in the same manner as the training data set; the images are resized, normalized, and augmentations are applied to the images. The trained model predicts the classification by obtaining the highest-probability class in classification problems, while in the segmentation problem, the model predicts the retinal relevant features in the image by creating a segmentation mask.

**Figure 5:**
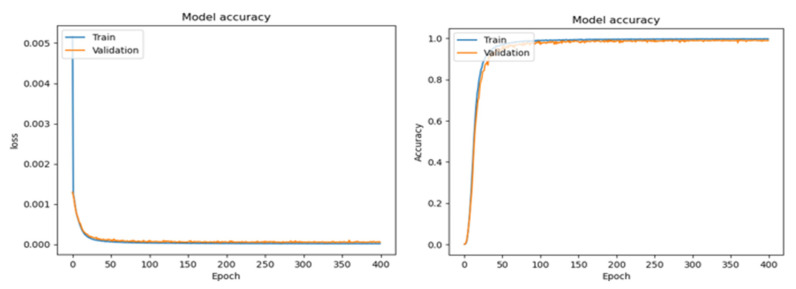
Testing and validation accuracy and loss over epochs with 50 K fundus images.

Figure 5 depict the training and validation loss (left) as well as the training and validation accuracy (right) of the proposed model over the epochs. In the case of the loss function, the training as well as the validation loss plummet sharply in the early epochs, signifying the efficient detection of the discriminative features. Subsequently, the graphs smooth and settle towards zero, signifying efficient optimization. The correlation between the training and the validation loss in the graph signifies that there is less overfitting, thereby ensuring that the model generalizes well on the novel instances. In the graph of the accuracy, the values of the training as well as the validation accuracy soar sharply in the initial epochs and settle towards 100%, signifying efficient feature extraction. Since the validation accuracy is closely correlated with the training accuracy, with minimal deviation, the graph signifies efficient generalization. The stable accuracy in the latter epochs of the graph signifies that the model is performing well even in the later phases of training. Overall, these curves validate the effectiveness of the proposed framework, showing rapid convergence, strong generalization, and stable learning, which are essential characteristics for reliable clinical deployment.

**Figure 6:**
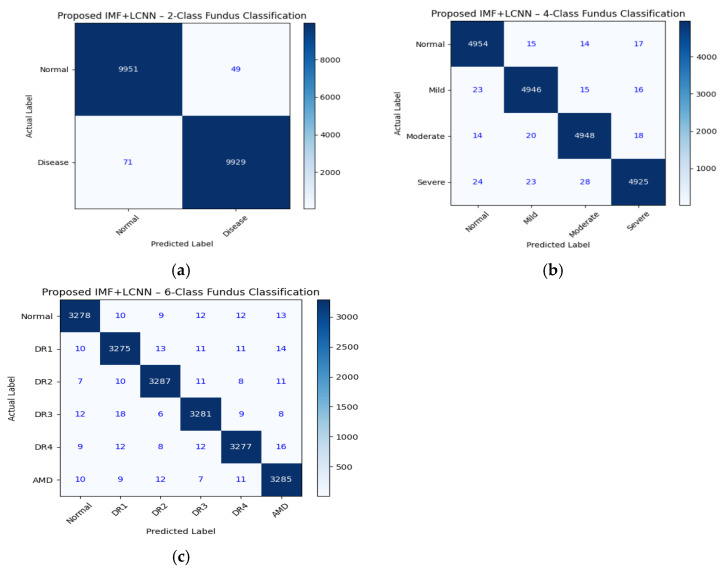
(**a**–**c**): (**a**) Representing Confusion Chart for the 2 labels for fundus classification. (**b**) Representing Confusion Chart for the 4 labels for fundus classification (**c**) Representing Confusion Chart for the 6 labels for fundus classification.

Figure 6a shows the confusion matrix for 2-class fundus image classification (Normal vs Diseased) using the proposed IMF+LCNN model. This shows that among the total number of tested images, 9951 are correctly identified as normal and 9929 are correctly identified as diseased, while only 49 normal images are mistakenly identified as diseased and 71 diseased images are mistakenly identified as normal images. This is associated with very low false-positive as well as false-negative values, which confirms the excellent discriminative ability of the model. Among the three clinical scenarios identified, the low value of the false-negative rate is more valuable, as it avoids the chances of mistakenly identifying a diseased case as a normal case, thereby ensuring patient safety and prevention of delayed treatment. Figure 6b above is the confusion matrix for 4-class classification tasks (Normal, Mild, Moderate, Severe), which capture the ordinal nature of disease severity stratification. The matrix shows dominance on the diagonal with correct predictions of 4954 for normal, 4946 for mild, 4948 for moderate, and 4925 for severe cases, while prediction errors are limited and restricted between adjacent classes, for instance, moderate and severe, and moderate and mild, due to visual similarities between progressive stages of the disease in the retina. It is, however, important to note that confusion between extreme classes (Normal and Severe), which is not allowed in ordinal regression, is not significant. Figure 6c shows the confusion matrix for 6-class fundus image classification corresponding to fine-grained disease staging (Normal, DR1, DR2, DR3, DR4, AMD). It is evident that the proposed model exhibits a large number of correct classifications for all classes, which are 3278 normal, 3275 DR1, 3287 DR2, 3281 DR3, 3277 DR4, and 3285 AMD samples. There are few misclassifications that are mostly confined to the neighbouring stages of diseases, ensuring proper training on learning progressions of diseases. Correct classification of varied stages of Diabetic Retinopathy with AMD showcases the efficiency of the developed framework in real-world applications.

## 4. Discussions

The experimental results presented above demonstrate the effectiveness of the proposed IMF+LCNN framework across binary, multi-class, and fine-grained disease classification tasks. In this section, a comparative analysis is carried out to contextualize the obtained performance with respect to existing state-of-the-art methods and to highlight the practical advantages of the proposed approach.

The relative performance of existing techniques and the proposed IMF+LCNN approach in terms of testing accuracy is shown quantitatively in Table 2a–c for low (5 K), medium (20 K), and large datasets of 50 K fundus images. Across low-, medium-, and large-scale datasets, the proposed IMF+LCNN framework consistently outperforms existing methods in terms of testing accuracy, recall, and F1-score. On smaller datasets, IMF-based enhancement plays a dominant role by improving signal quality and mitigating illumination-related variability, leading to substantial gains in recall and precision. As dataset size increases, the contribution of the lightweight architecture becomes more prominent, enabling stable generalization while avoiding overfitting observed in deeper or GAN-based models.

The consistently high recall values achieved by the proposed framework across all dataset scales indicate reliable detection of pathological cases, while high precision values reduce unnecessary referrals. These findings confirm that the proposed IMF+LCNN approach achieves both **statistical superiority and clinical relevance**.

### Achievements of the Proposed IMF+LCNN Framework

The proposed IMF+LCNN framework achieves multiple critical objectives through its deliberate architectural and preprocessing design choices. By combining IMF-based adaptive signal decomposition with a lightweight CNN architecture, the framework achieves high diagnostic accuracy across binary, multi-class, and fine-grained disease classification tasks. The consistently low false-negative rates validate its clinical reliability, while reduced computational complexity ensures scalability and suitability for real-world deployment.

## 5. Conclusions

In this work, the authors have developed a skilled and practical IMF+LCNN framework that is universally effective for the automated analysis of retinal fundus images. The framework, which uses Intrinsic Mode Function adaptive signal decomposition in combination with the lightweight convolutional neural network, is successfully able to tackle significant challenges in the retinal imaging procedure. In the above sections, the authors have experimented in three ways: the 2-class, 4-class, and 6-class classifications. In the 2-class test, the framework is able to successfully differentiate between normal and unhealthy retinal images. Hence, the framework is very efficient for the preliminary screening process. In the 4-class test, the framework is able to successfully specify the stages of the unhealthy retinal images, due to which the framework is very helpful in the clinical process. The 6-class test is also successful in the sense that the framework is not able to misclassify closely related stages of unhealthy retinal images, such as the stages of Diabetic Retinopathy and the stages of age-related macular degeneration. The low values of the numbers of false negatives in all tests also confirm the clinical correctness of the framework. The framework is very competent, efficient, and easily interpretable.

***Scope:***

Future studies will aim at extending the above proposed framework towards the incorporation of multimodal retinal imaging studies such as the combination of fundus images and optical coherence tomography (OCT). Furthermore, cross-dataset and multi-centre validation studies will also be pursued for further confirmation of the generalizability and regulatory readiness of the proposed system. Incorporating the above-mentioned explainable AI methodologies and the potential for data based on longitudinal patient studies will also enable the confirmation of disease progression analysis and clinical explainability for the above-proposed system.

## Figures and Tables

**Table 1:** Summary of related work.

Ref No.	Authors	Key Contributions	Main Findings	Identifies Research Gap
[1]	H. Lei et al.	Proposed an unsupervised domain adaptation framework using image synthesis and feature alignment for joint optic disc and cup segmentation	Demonstrated improved segmentation performance across datasets with domain variability	Focused only on segmentation; does not address disease classification for real time deployment
[3]	M.S. Junayed et al.	Developed CataractNet, a CNN-based automated cataract detection model for fundus images	Achieved high accuracy for cataract detection and showed feasibility of automated screening	Limited to single disease, lacks adaptive feature enhancement and generalization across multiple retinal conditions
[6]	X.Li et al.	Introduced a rotation based self-supervised learning framework exploiting fundus image symmetry	Improved diagnostic accuracy using unlabeled data and enhanced model	Relies on heavy backbone networks; complexity and deployment constraints are not addressed
[11]	F Chen et al.	Proposed a dual path, multi-scale attention network for retinal disease classification using ultra-wide field images	Achieved improved lesion detection and classification accuracy using attention mechanisms	Uses complex deep architectures, lacks lightweight design and adaptive signal decomposition for noise suppression
[18]	M. Shafiq et al.	Proposed Dual Eye-Feature Net for multimodal ophthalmic image classification using fundus and OCT images	Improved classification performance through feature transfer between modalities	Requires multi-modal data (Fundus OCT), limiting applicability in fundus-only screening scenarios

**Table 2:** Performance comparison across dataset scales (low, medium, and large fundus datasets). (**a**) Performance comparison across dataset scales: low dataset (5 k fundus images). (**b**) Performance comparison across dataset scales: medium dataset (20 k fundus images). (**c**) Performance comparison across dataset scales: large dataset (50 k fundus images).

**(a)**					
**ALGORITHMS WITH FUNDUS 20 K**	**ACCURACY (TRAINING)**	**ACCURACY (TESTING)**	**PRECISION**	**RECALL**	**F1-SCORE**
**CNN [12]**	88.40	85.10	86.20	82.75	84.44
**LSTM [7]**	87.95	80.40	77.10	83.60	80.23
**Ensemble (CNN) [5]**	79.30	82.10	83.55	81.20	82.36
**GAN [6]**	97.80	96.90	95.85	95.60	95.72
**ResNet [18]**	84.60	86.20	86.75	83.40	85.04
**IMF [21]**	98.20	97.60	96.45	95.80	96.12
**Proposed IMF+LCNN**	98.90	98.35	98.20	97.85	98.02
**(b)**					
**ALGORITHMS WITH FUNDUS 20 K**	**ACCURACY (TRAINING)**	**ACCURACY (TESTING)**	**PRECISION**	**RECALL**	**F1-SCORE**
**CNN [12]**	90.50	88.25	89.36	85.14	86.25
**LSTM [7]**	90.63	82.25	79.29	85.64	81.75
**Ensemble (CNN) [5]**	81.25	84.25	86.41	83.64	83.58
**GAN [6]**	95.63	91.98	94.52	97.71	96.89
**ResNet [18]**	86.36	88.29	88.49	85.96	87.28
**IMF [21]**	98.95	94.41	92.25	91.36	95.21
**Proposed IMF+LCNN**	98.40	97.40	95.01	95.87	96.73
**(c)**					
**ALGORITHMS WITH FUNDUS 20 K**	**ACCURACY (TRAINING)**	**ACCURACY (TESTING)**	**PRECISION**	**RECALL**	**F1-SCORE**
**CNN [12]**	92.30	90.15	90.85	88.40	89.61
**LSTM [7]**	91.75	85.60	82.40	87.90	85.05
**Ensemble (CNN) [5]**	84.90	87.40	88.20	85.75	86.96
**GAN [6]**	99.05	93.60	93.80	95.95	94.87
**ResNet [18]**	88.95	90.40	90.10	88.65	89.37
**IMF [21]**	94.20	92.90	91.95	90.20	91.57
**Proposed IMF+LCNN**	99.65	94.58	91.30	97.05	95.17

## Data Availability

The raw data supporting the conclusions of this article will be made available by the author on request.

## Abbreviations

The following abbreviations are used in this manuscript:

IMF	Intrinsic Mode Function
EMD	Empirical Mode Decomposition
LCNN	Light Convolutional Neural Network
ResNet	Residual Neural Network
DL	Deep Learning
ML	Machine Learning
DR	Diabetic Retinopathy
GANs	Generative Adversal Networks
UDA	Unsupervised Domain Adaptation
ReLU	Rectified Linear Unit
CLAHE	Contrast Limited Adaptive Histogram Equalization.
AUC ROC	Area Under the Curve Receiver Operating Characteristics
